# Treatment of Arachnoid Cyst With Spontaneous Hemorrhage With Atorvastatin

**DOI:** 10.3389/fphar.2019.01343

**Published:** 2019-11-22

**Authors:** Bei Liu, Chao Wang, Yan Qu

**Affiliations:** Department of Neurosurgery, Tangdu Hospital, Fourth Military Medical University, Xi’an, China

**Keywords:** atorvastatin, children, arachnoid cyst, subdural hematoma, neurosurgery

## Abstract

As one of the common neurological diseases, pediatric middle fossa arachnoid cysts(MFACs) can develop intracystic hemorrhage and subdural hematoma. Risk factors for pediatric arachnoid cyst rupture/hemorrhage is very complicated in mechanism. Although surgery is the first choice for children with MFACs and subdural hematoma, the rate of recurrence of the subdural hematoma is very high after 1 or more surgeries. Atorvastatin has proven to be a bold and safe choice in the management of subdural hematoma with mild symptoms. The present study has described a 7-year-old child with a recurrent rupture of arachnoid cyst develops into a subdural hematoma. We demonstrate that atorvastatin is safe and effective in pediatric patient who has failed surgical treatment of middle fossa arachnoid cyst and subdural hematoma. The patient received atorvastatin monotherapy, once daily for the first week, with an initial dose of 5 mg, followed by 10 mg once daily for 7 weeks. In the third month after the initial treatment, the neurological function recovered, and the hematoma completely resolved. This case report supports the concept that atorvastatin can promote the absorption of subdural hematoma.

## Introduction

Intracranial arachnoid cysts are benign lesions that commonly occur in children. Although most arachnoid cysts are asymptomatic, a small number of patients experience a spontaneous cyst rupture or hemorrhage which can be life-threatening in severe cases. Subdural hematoma caused by repeated rupture of cysts may be associated with inflammation, angiogenesis, local coagulation abnormalities, recurrent microbleeds, and local secretions (Holl et al., 2018). Therefore, considering the pathophysiological mechanisms, the mortality and high morbidity associated with craniotomy, drug therapy might be a beneficial alternative for patients. Atorvastatin has proven to be a bold and safe choice in the management of subdural hematoma in adults with mild symptoms (Wang et al., 2014). In this case, we used atorvastatin to treat a child with repeated subdural hematomas due to ruptured arachnoid cyst.

## Case Presentation

A 7-year-old male child (weight: 21 kg) was diagnosed with a large left-side MFAC by a CT scan of the head in community hospitals. There was no history of traumas. After 1 month, the patient developed severe headaches, dizziness, nausea, and vomiting. An MRI scan revealed a left temple lobe arachnoid cyst and a left frontal and temporal-parietal subdural hematoma (**Supplementary Figure 1A** and **B**). The patient was treated with cyst cerebral cistern ostomy, and the subdural hematoma was removed under neuroendoscope in our hospital (**Supplementary Figure 1C** and **D**). The patient recovered well; any postoperative headaches dissipated within X days after the surgery. No neurological dysfunction was observed.

However, 26 days after surgery, the patient experienced headaches and repeated nausea and vomiting. A CT scan of the head showed a left subdural hematoma in the frontal and temporal-parietal regions (**Supplementary Figure 1E** and **F**). A burr-hole craniotomy (BHC) was performed in the emergency department to remove the hematoma. The postoperative symptoms of the patient’s caused by cranial hypertension related to the subdural hematoma resolved and the patient recovered well (**Supplementary Figure 1G** and **H**).

**Figure 1:**
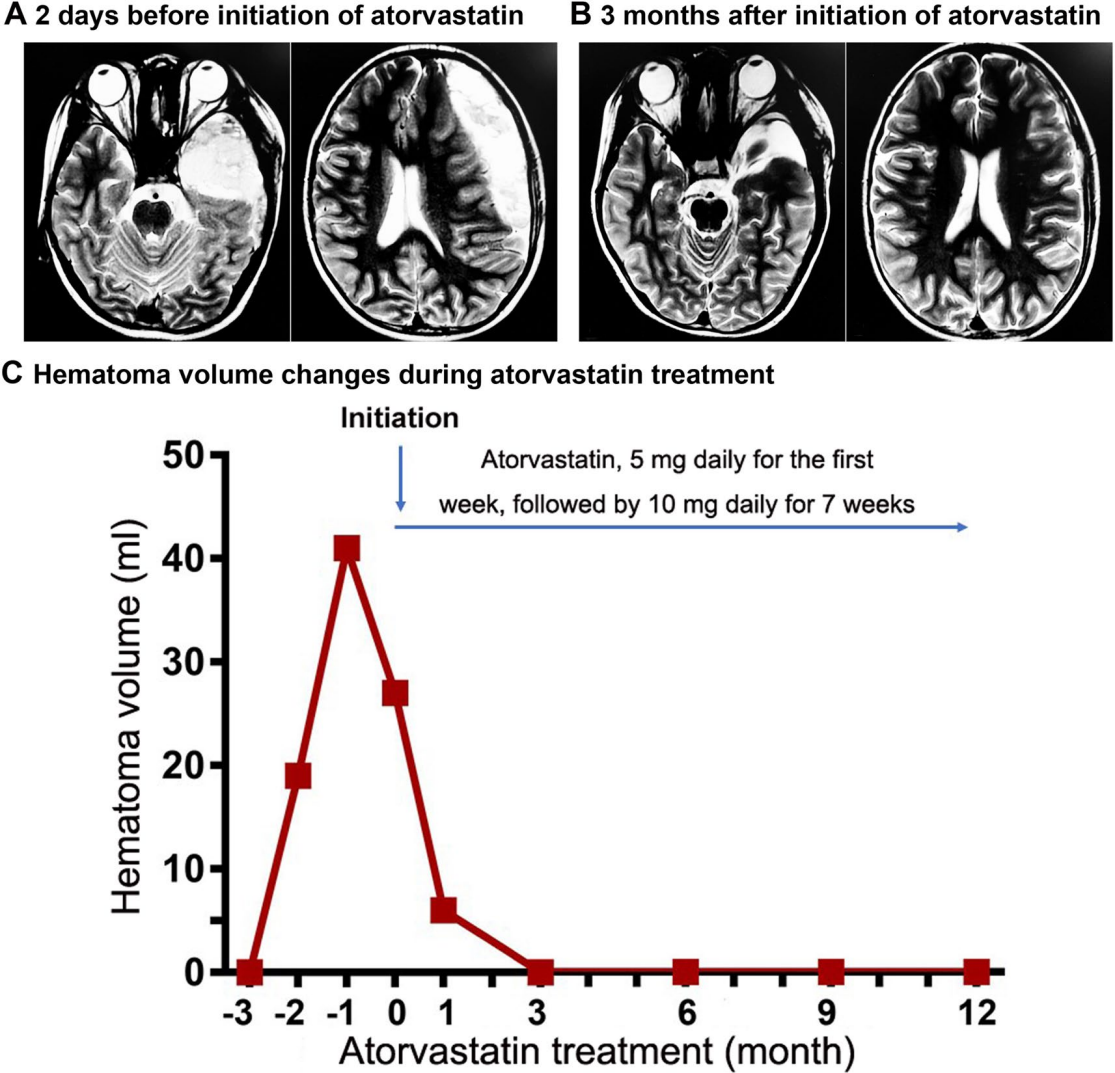
MRI of the head and hematoma volume changes during atorvastatin Treatment. The head MRI scans were performed 2 days before the initiation of atorvastatin therapy **(A)** and 3 months after therapy was started **(B)**. Images are from T2-weighted sequencing. **(C)** shows a gradual decrease in hematoma volume after atorvastatin treatment **(** **Supplementary Figures 1–6** **)**.

On the 27th day after surgery, the patient’s headaches reemerged along with double vision, and mild strabismus in the left eye. An MRI examination identified postoperative changes to the left temple lobe arachnoid cyst, and formation of a left frontal- temporal subdural hematoma (**Figure 1A**). General physical and neurological examinations revealed no abnormalities. Laboratory findings were within normal limits except for an alkaline phosphatase of 239 U/L.

The patient received atorvastatin monotherapy once daily for eight weeks, with an initial dose of 5 mg daily for the first week, followed by 10 mg a day for 7 weeks. Follow-up monitoring was done monthly for 12 months after the initial atorvastatin treatment (**Figure 1C**). Laboratory tests and physical examination were thoroughly conducted and MRI follow-up was performed regularly. The patient experienced improvements in symptoms and a significant decrease in hematoma volume within the first month of the treatment (**Supplementary Figure 3A** and **B**). By the third month after the initial treatment, neurological function had fully recovered and the hematoma had completely resolved (**Figure 1B**).

## Discussion

Intracranial arachnoid cysts are benign lesions that commonly occur in children. Intracranial arachnoid cysts account for about 1% of children’s intracranial lesions, 90% of which are identified during a routine screening, primarily (60% of the time) in the middle cranial fossa (Al-Holou et al., 2010). Patients with arachnoid cysts with a diameter of ≥5 cm that have recently experienced a head trauma are at risk for cyst rupture or bleeding. A cyst rupture involving a relatively large blood vessel can lead to a subdural hematoma, while a rupture in a relatively hypovascular brain region may lead to subdural cerebrospinal fluid collection only.

This patient suffered spontaneous rupture of his MFACs, producing subdural hematoma. Rupture of the cyst produces one or more tears in the cyst walls, which directs fluid into both the subdural and subarachnoid spaces. MRI showed a line-like high-density shadow in the hematoma of the subdural hematoma, which is expected to be a separate hematoma capsule. BHC is not suitable for septal subdural hematoma. Considering the pathophysiological mechanisms, the mortality and high morbidity associated with craniotomy, drug therapy might be a beneficial alternative for this patient. The results of mannitol trials for the treatment of subdural hematoma are inconsistent. However, studies have shown that oral atorvastatin can significantly reduce the risk of recurrence of adult subdural hematoma. Therefore, oral atorvastatin is the most suitable treatment for this patient.

Repeated subdural hematoma after arachnoid rupture may be caused by several factors including: inflammation, angiogenesis, local coagulation abnormalities, thick hematoma envelope, impaired brain parenchyma swelling, inability to close the subdural space, and rebleeding of arachnoid cysts. Due to the mortality, morbidity, and recurrence postoperative hematoma rates and considering the pathophysiological mechanism of subdural hematoma in arachnoid cysts, oral drug therapy for treatment of this patient is worth investigating.

Atorvastatin has proven to be a bold and safe choice in the management of subdural hematoma with mild symptoms. Atorvastatin is one of the most effective statins, with fewer side effects (such as myopathy and hepatotoxicity) compared to other statins. Recent studies have found that atorvastatin reduces formation of subdural hematomas by enhancing angiogenesis and reducing the inflammatory response in rats (Li et al., 2014; Wang et al., 2016). One potential mechanism of action for Atorvastatin includes up-regulation of BDNF expression, which may contribute to functional recovery after stroke (Ludka et al., 2017). Geifman et al. found that atorvastatin can significantly reduce the incidence of cognitive impairment in patients with Alzheimer’s disease (Geifman et al., 2017); thus, Atrovastin may be especially critical for children who are unresponsive to surgical treatment of repeated subdural hematoma.

Wang et al. reported that perioperative atorvastatin adjuvant therapy reduces recurrence rates and does not increase morbidity and mortality in adult patients with subdural hematoma undergoing BHC (Tang et al., 2018). Another randomized, placebo-controlled, double-blind phase II clinical trial reported that atorvastatin reduces hematoma, and improves neurological function and quality of life; the effect persisted for a follow-up period of 16 weeks in nonsurgical patients with subdural hematoma (Jiang et al., 2018). In addition, Huang et al. found that atorvastatin combined with dexamethasone is a safe and effective option for the treatment of pediatric patients with recurrent subdural hematoma (Huang et al., 2019).

## Conclusion

This case report supports the concept that atorvastatin can promote the absorption of subdural hematoma. Based on this experience, we believe that clinicians with similar child patients should consider treatment with atorvastatin.

## Data Availability Statement

All datasets generated for this study are included in the article/**Supplementary Material**.

## Ethics Statement

The studies involving human participants were reviewed and approved by Ethics Committee of Tangdu Hospital of the Fourth Military Medical University. Written informed consent to participate in this study was provided by the participants’ legal guardian/next of kin. Written informed consent was obtained from the minor(s)’ legal guardian/next of kin for the publication of any potentially identifiable images or data included in this article.

## Author Contributions

BL and CW cared for the patient. CW and BL collected and analyzed the data. BL and YQ wrote the manuscript. YQ reviewed the paper. BL and CW provided the image.

## Conflict of Interest

The authors declare that the research was conducted in the absence of any commercial or financial relationships that could be construed as a potential conflict of interest.
