# Supplementary material for: Treatment of Arachnoid Cyst With Spontaneous Hemorrhage With Atorvastatin
Source: Front Pharmacol. 2019 Nov 22;10:1343. doi: 10.3389/fphar.2019.01343 (PMC6884023; doi:10.3389/fphar.2019.01343)
Supplement: Supplementary file 1 [file DataSheet_1.docx]

Clinical experience in children with MFACs and subdural hematoma

As one of the common neurological diseases, pediatric middle fossa arachnoid cysts（MFACs) can develop intracystic hemorrhage and subdural hematoma. Risk factors for pediatric arachnoid cyst rupture/hemorrhage is very complicated in mechanism. Although surgery is the first choice for children with MFACs and subdural hematoma, the rate of recurrence of the subdural hematoma is very high after 1 or more surgeries. Atorvastatin has proven to be a bold and safe choice in the management of subdural hematoma with mild symptoms.

In our pediatric neurosurgery center, the child with MFAC and subdural hematoma were successfully treated with atorvastatin.

The case is described in detail in the article.


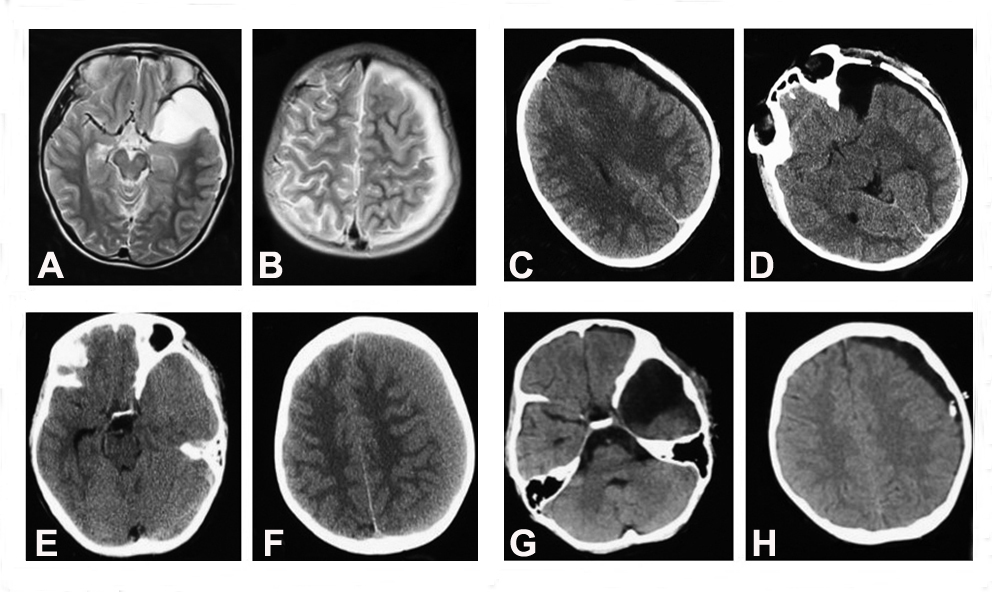


Supplement Fig 1. Head MRI showed a 7-year-old male child with intracystic hemorrhage (Supplement Fig 1A) and subdural hematoma (Supplement Fig 1B); In Figures C and D, a head CT scan showed postoperative intracranial changes in cyst cerebral cistern ostomy; In Figures E and F, a head CT scan showed relapsing subdural hematoma after cyst cerebral cistern ostomy; In Figures G and H, a head CT scan showed postoperative intracranial changes in burr-hole craniotomy.


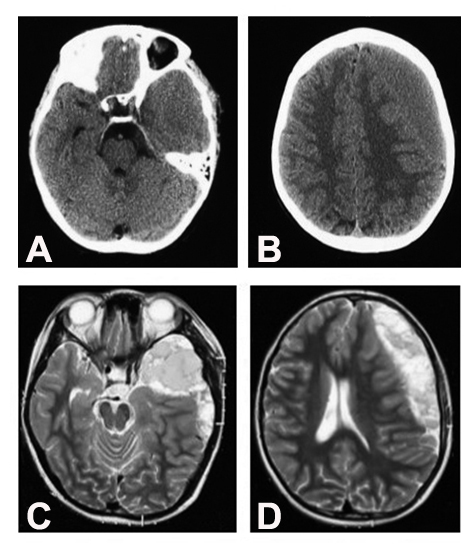


Supplement Fig 2. In Figures A and B, a head CT scan showed relapsing intracystic hemorrhage (Supplement Fig 2A) and subdural hematoma (Supplement Fig 2B); In Figures C and D, Head MRI showed left temple lobe arachnoid cyst postoperative changes, left frontal subdural hematoma, separated formation.


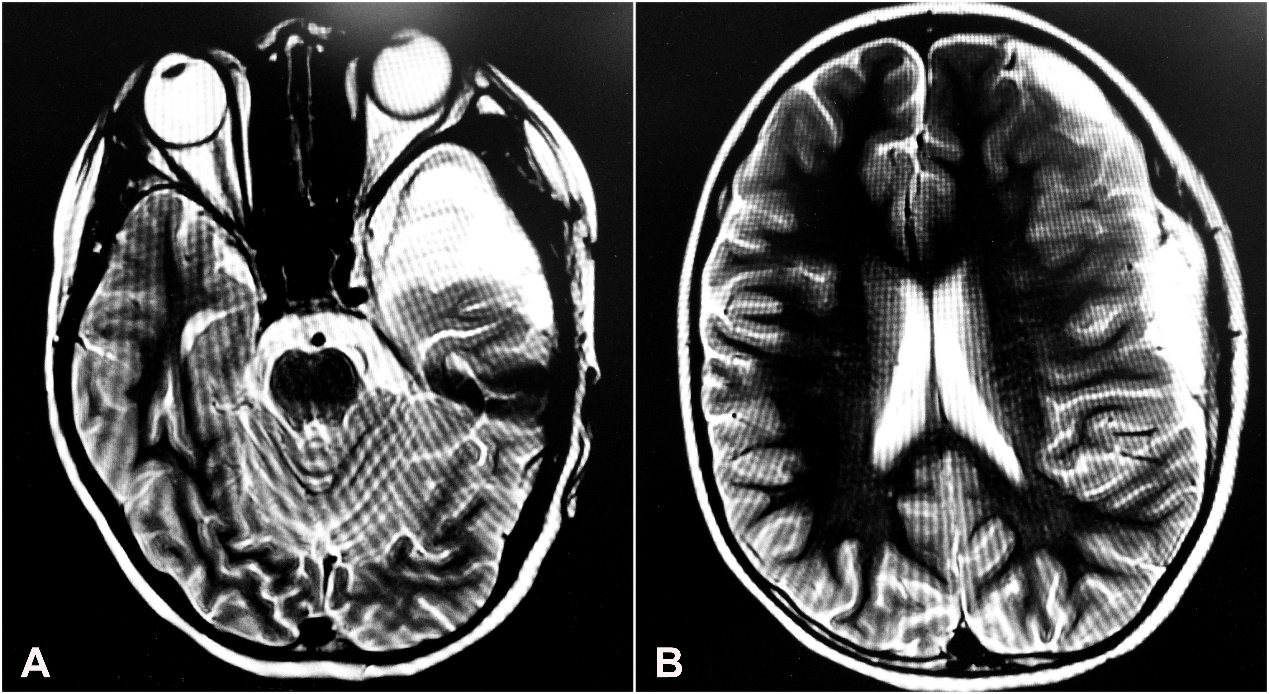


Supplement Fig 3. In Figures A and B, head MRI showed a significantly decreased in hematoma volume in the first month of atorvastatin treatment.


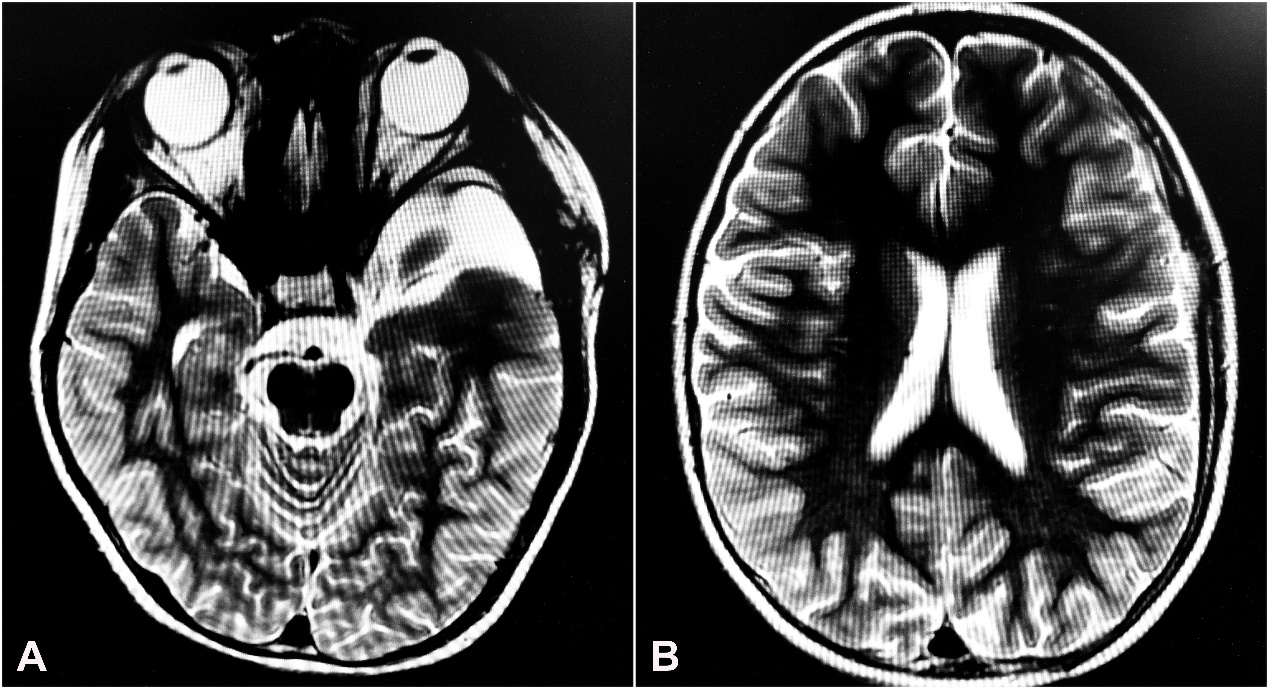


Supplement Fig 4. In Figures A and B, head MRI showed the hematoma completely resolved in the third month after the initial treatment.
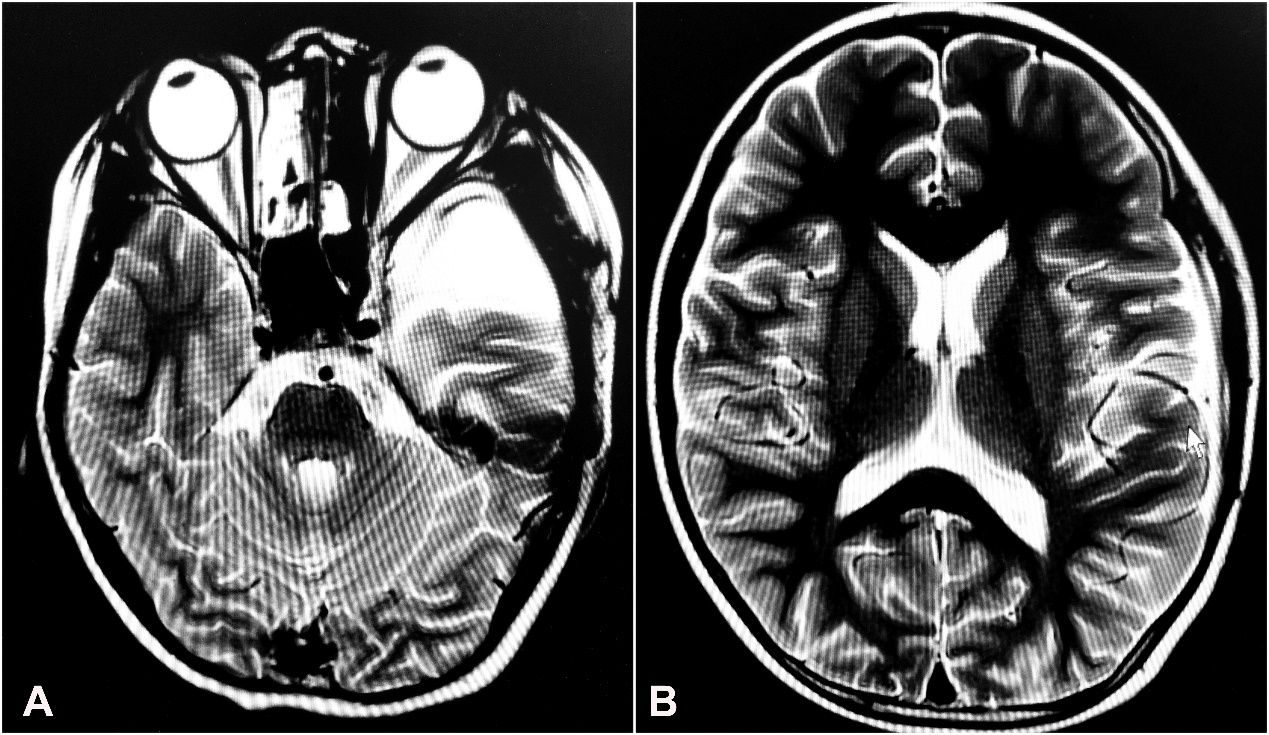


Supplement Fig 5. In Figures A and B, head MRI showed no recurrence of hematoma in the sixth month after the initial treatment.


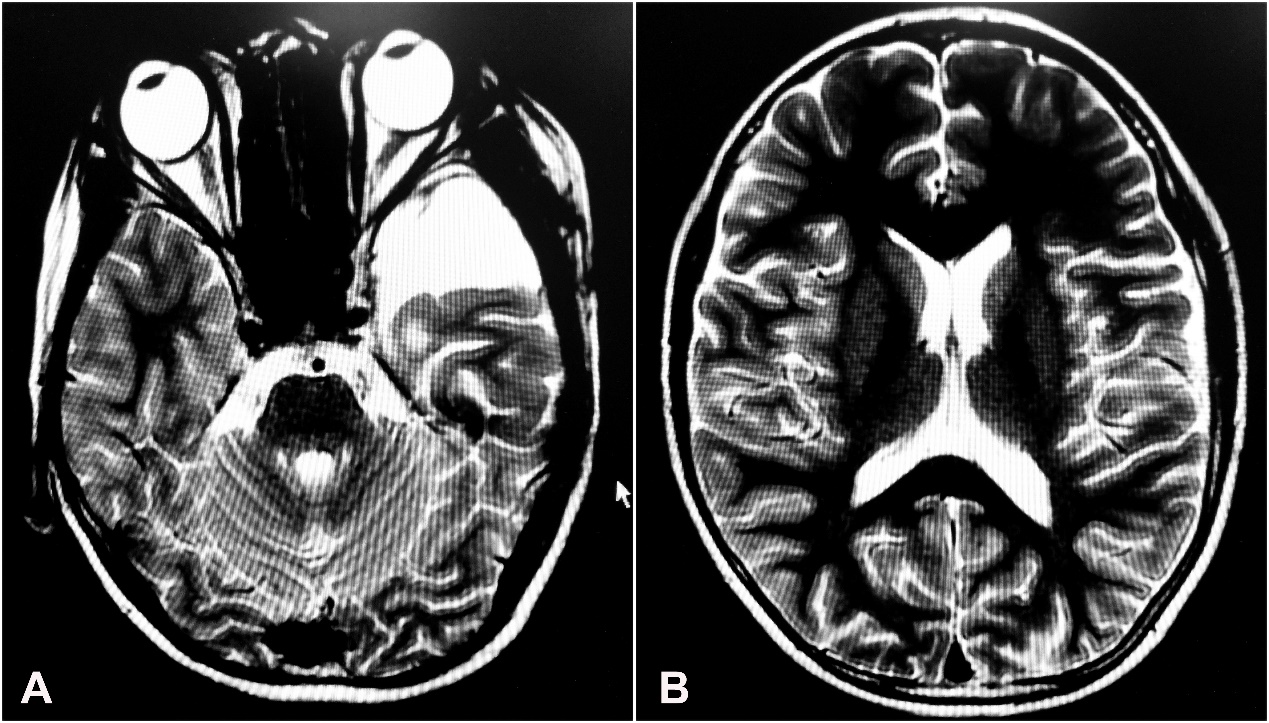


Supplement Fig 6. In Figures A and B, head MRI showed no recurrence of hematoma in the first year after the initial treatment.
